# Mir-150-5p distinguishes acute pulmonary embolism, predicts the occurrence of pulmonary arterial hypertension, and regulates ox-LDL-induced endothelial cell injury

**DOI:** 10.1186/s41065-024-00333-z

**Published:** 2024-09-10

**Authors:** Yue Wu, Xin Sun, Guangqiang Cui, Shu Wang

**Affiliations:** 1 Department of Vascular Surgery, Zibo Central Hospital, Zibo, 255020 Zibo China; 2 Department of Cardiothoracic Surgery, Zibo Central Hospital, Zibo, 255020 Zibo China; 3 Department of Respiratory and Critical Care Medicine, Zibo Central Hospital, No. 54, Gongqingtuan West Road, Zhangdian District, Zibo, 255020 Shandong China

**Keywords:** Venous thromboembolism, Acute pulmonary embolism, Pulmonary arterial hypertension, Diagnostic biomarker, Inflammation, Oxidative stress

## Abstract

**Background:**

Acute pulmonary embolism (APE) is a major type of venous thromboembolism (VTE) with a high risk of mortality and disability. There is a lack of biomarkers for APE to indicate deteriorating development and predict adverse outcomes. This study evaluated the significance of miR-150-5p in APE aiming to explore a novel potential biomarker for APE.

**Methods:**

The study enrolled APE (*n* = 137) and deep wein thrombosis (DVT, *n* = 67) patients and collected plasma samples from all study subjects. The expression of miR-150-5p was analyzed by PCR and its significance in screening APE and pulmonary arterial hypertension (PAH) was assessed by receiver operating curve (ROC) and logistic analyses. The study established oxidized low-density lipoprotein (ox-LDL)-induced human venous endothelial cells (HUVECs). Through cell transfection combined with cell counting kit-8 (CCK8), flow cytometry, and enzyme-linked immunosorbent assay (ELISA), the effect of miR-150-5p on ox-LDL-induced HUVEC injury was evaluated.

**Results:**

Significant downregulation of miR-150-5p was observed in the plasma of APE patients compared with DVT patients (*P* < 0.0001). The plasma miR-150-5p levels in APE patients occurred PAH was much lower than in patients without PAH (*P* < 0.0001). Reducing miR-150-5p distinguished APE patients from DVT patients (AUC = 0.912) and was identified as a risk factor for the occurrence of PAH in APE patients (OR = 0.385, *P* = 0.010). In HUVECs, oxidized low-density lipoprotein (ox-LDL) caused inhibited cell proliferation, enhanced apoptosis, increased pro-inflammatory cytokines, reactive oxygen species (ROS), malondialdehyde (MDA), and decreased superoxide dismutase (SOD). Overexpressing miR-150-5p could promote proliferation, inhibit apoptosis, and alleviate inflammation and oxidative stress of ox-LDL-treated HUVECs.

**Conclusions:**

Downregulated plasma miR-150-5p served as a diagnostic biomarker for APE and predicted the predisposition of PAH in APE patients. Overexpressing miR-150-5p could alleviate ox-LDL-induced endothelial cell injury in HUVECs.

**Supplementary Information:**

The online version contains supplementary material available at 10.1186/s41065-024-00333-z.

## Introduction

Acute pulmonary embolism (APE) possesses complex pathological and physiological characteristics, and hypercoagulability, endothelial dysfunction, and hemodynamic changes have been considered the main pathogenesis of APE [1]. Additionally, the severity of disease conditions is also associated with factors such as the size and number of thrombi, the interval of embolization, the rate of thrombus dissolution, and complications [2]. Although increasing attention has been paid to APE, there is still a high rate of misdiagnosis and missed diagnosis due to the lack of effective diagnostic methods. The diagnosis of APE is mainly through imaging examination and related markers, such as D-dimer (D-D) [3, 4]. However, the high cost of imaging examination and low specificity of D-D limit their application. Therefore, there is an urgent need to explore novel biomarkers assisting the diagnosis of APE and improving patients’ prognosis.

Recent studies have noticed the significance of microRNAs (miRNAs) in regulating the development of various human diseases, including venous thrombosis [5, 6]. Circulating miRNAs have the advantages of easy collecting, strong stability, high specificity, and repeatability and have been identified as reliable diagnostic biomarkers for APE, and several miRNAs were demonstrated to be associated with the onset, prognosis, and disease-related processes of venous thromboembolism, such as miR-205-5p, miR-195-5p, miR146a, miR-149, miR-499, and miR-196a2 [7, 8]. A previous study observed the differential expression of miR-150-5p in the blood of PE patients, and miR-150-5p was revealed to show a negative correlation with the recurrence of venous thromboembolism [9–11]. miR-150-5p was also identified as a biomarker for the diagnosis and prognosis of chronic obstructive pulmonary disease (COPD), which is a critical risk factor for APE [12]. Additionally, miR-150-5p could regulate the angiogenic capability of endothelial progenitor cells and endothelial inflammation, which is also associated with APE progression [13, 14]. Therefore, miR-150-5p was hypothesized of great potential in APE, but there were no data available to confirm its significance in APE.

Inflammation and oxidative stress are the main pathways that induce endothelial cell injury in human umbilical vascular endothelial cells (HUVECs), which would further promote the progression of APE to a severe direction. Previous studies have employed oxidized low-density lipoprotein (ox-LDL)-induced HUVECs to disclose the regulatory effect of miRNAs on related disease progression. Hence, the mechanism underlying the regulation of endothelial cell injury by miR-150-5p was also investigated with the employment of ox-LDL-induced HUVECs to clear its involvement in APE progression.

In this study, the expression and significance of miR-150-5p in APE were evaluated with the enrollment of APE patients and deep vein thrombosis (DVT) patients. Deeply, to reveal the mechanism underlying the function of miR-150-5p, the expression and regulatory effect of miR-150-5p on vascular endothelial cell injury were investigated, aiming to explore a potential biomarker for the diagnosis and progression of APE.

## Methods

### Patients

137 APE and 67 DVT patients were enrolled from Zibo Central Hospital from January 2020 to December 2022. The flowchart for patient enrollment is shown as Figure S1. The age, gender composition, blood pressure indexes, and blood lipid indixes were matched between the two groups during the enrollment. The inclusion criteria for the two groups were as follows:

APE patients were primarily diagnosed by computed tomographic pulmonary angiography without the complication of DVT and had never received treatments before their enrollment. Patients with following complications were excluded: (1) inflammatory diseases; (2) ischemic diseases; (3) severe dysfunction of the liver and kidney; (4) chronic pulmonary hypertension and other pulmonary diseases.

DVT patients possessed matched age and gender composition with APE patients without the complication of PE. The study had been approved by the Ethics Committee of Zibo Central Hospital (No. 2019081), and all participants had provided informed consent.

### Sample collection

Fasting venous blood (5 mL) was collected from all participants on the next morning of their enrollment. Blood samples were collected into the anticoagulation tubes, and plasma was isolated by centrifugating at 3500 rpm for 10 min. Plasma samples were stored at -80 °C for the following analyses.

### Cell culture and grouping

Human umbilical vascular endothelial cells (HUVECs) obtained from ATCC were employed for establishing an injured cell model. Cells were incubated with DMEM culture medium supplemented with 10% FBS at 37 °C with 5% CO_2_.

Cells were transfected with miR-150-5p mimic or its negative control generated by Riobo Biotechnology Ltc. (China) using Lipofectamine 2000 (Invitrogen, USA) to assess the regulatory effect of miR-150-5p on HUVEC cell injury. Transfected cells were available for cell modeling after 48 h of cell transfection. Cells were treated with 150 µg/mL ox-LDL for 24 h to induce cell injury. Untransfected cells were treated with ox-LDL (Yuanye Biotechnology, China) after the confluence reached 75%. Cells without ox-LDL treatment and cell transfection were set as the control group.

### Total RNA extraction

Plasma samples or cells were mixed with TRI reagent BD (TB-126, Molecular Research Center, USA) and stood for 5 min at room temperature. Then, 200 µL chloroform was added and shaken for 15 s. After centrifugation at 12,000 rpm for 15 min, the water phase was transferred to new RNase-free EP tubes and mixed with 500 µL pre-cold isopropanol. The mixture was stood at room temperature for 10 min and then centrifugated at 12,000 rpm for 8 min at 4 °C. The precipitate was further washed with 75% ethanol and dried in the super clean table. The purity and concentration of extracted RNA were evaluated by the ratio of OD260/280 using NanoDrop 2000 (Thermo Fisher Scientific, USA).

### Real-time quantitative PCR

Reversed transcription was performed with the extracted RNA using the TaqMan MicroRNA Reverse Transcription Kit (Applied Biosystem, USA). Then, quantitative PCR was conducted with the TaKaRa PCR kit (Takara Bio, Japan) on the LightCycler 480 Real-Time PCR System (Roche, Australia). The relative expression of miR-150-5p was calculated by the 2^−ΔΔCT^ method with cel-miR-39 as the internal reference.

### Cell proliferation assay

Cells were seeded into 96-well plates supplied with a completed culture medium at 37 °C with 5% CO_2_. The incubation was continued for 0, 24, 48, 72, and 96 h, and then CCK8 reagent was added to each well. Another 4-h incubation was performed, and the plates were measured with a microplate reader at 450 nm.

### Cell apoptosis assay

Cells were lysed with pancreatase (ETDA-free) and washed with pre-cold PBS buffer. Cells were resuspended with Binding Buffer and incubated with Annexin V-FITC in the dark for 15 min. Then, cells were stained by PI for 5 min. After mixing with the Binding Buffer, cell apoptosis was measured with a flow cytometer in the dark.

### Oxidative stress evaluation

Oxidative stress in HUVECs was evaluated by the levels of reactive oxygen species (ROS), superoxide dismutase (SOD), and malondialdehyde (MDA).

ROS was detected with a DCHF-DA probe incubated in the dark for 10 min. The fluorescence intensity was analyzed by a fluorescence microplate reader (Bio Tek Synergy H4, USA).

Cells were incubated in 6-well plates at a density of 1 × 10^5^ cells/well and collected after 24 h. Cell lysis was employed for the analyses of SOD and MDA. The levels of SOD and MDA were measured with corresponding kits (Jiancheng Bioengineering, China) according to the instruments.

$$\begin{array}{l}{\rm{MDA}}\,\left( {{\rm{nmol/mg}}} \right)\,{\rm{ = }}\,\left[ {\left( {{\rm{O}}{{\rm{D}}_{{\rm{sample}}}}{\rm{ - O}}{{\rm{D}}_{{\rm{control}}}}} \right){\rm{/}}\left( {{\rm{O}}{{\rm{D}}_{{\rm{standards}}}}{\rm{ - O}}{{\rm{D}}_{{\rm{blank}}}}} \right)} \right]\\{\rm{ \times concentration}}\,{\rm{of}}\,{\rm{standards/protein}}\,{\rm{concentration}}\end{array}$$

$$\begin{array}{l}{\rm{SOD}}\,\left( {{\rm{U/mg}}} \right)\,{\rm{ = }}\,{\rm{inhibition}}\,{\rm{rate/50\% }}\\{\rm{ \times dilution}}\,{\rm{ratio/protein}}\,{\rm{concentration}}\end{array}$$

$$\begin{array}{l}{\rm{inhibition}}\,{\rm{rate}}\,\left( {\rm{\% }} \right)\,{\rm{ = }}\,\left[ {\left( {{{\rm{A}}_{{\rm{control}}}}{\rm{ - }}{{\rm{A}}_{{\rm{control blank}}}}} \right){\rm{ - }}\left( {{{\rm{A}}_{{\rm{sample}}}}{\rm{ - }}{{\rm{A}}_{{\rm{sample blank}}}}} \right)} \right]\\{\rm{/}}\left( {{{\rm{A}}_{{\rm{control}}}}{\rm{ - }}{{\rm{A}}_{{\rm{conctrol blank}}}}} \right){\rm{ \times 100\% }}\end{array}$$

### Enzyme-linked immunosorbent assay

Cells were centrifugated at 1500 rpm for 5 min, and the supernatant was mixed with antibodies of TNF-α, IL-1β, and IL-6 (Beyotime Biotechnology, USA). After incubating for 1 h, streptavidin labeled with horseradish peroxidase (Invitrogen, USA) was added and incubated at room temperature for 20 min. Finally, the solution was mixed with TMB substrate solution (Thermo Fisher, USA) and incubated in the dark for 20 min. The reaction was terminated by EDTA, and the absorbance at 450 nm was measured by a microplate reader.

### Statistical analyses

Data were analyzed using SPSS 23.0 software and GraphPad Prism 9.0 software. Cell experiments were performed with three independent repeats. Difference comparison was performed with a student’s t-test or one-way ANOVA (*P* < 0.05). ROC analysis was conducted to evaluate the significance of miR-150-5p in distinguishing APE patients. Logistic regression analysis was employed to evaluate the risk factors for APE and pulmonary arterial hypertension (PAH) in DVT patients and APE patients, respectively. The correlation between miR-150-5p and patients’ clinicopathological features was assessed by Pearson correlation analysis (*r* > 0.5, *P* < 0.05).

## Results

### Baseline information of DVT and APE patients

DVT patients included 35 males and 32 females with an average age of 60.48 ± 8.77 years, while APE patients included 72 male patients and 65 female patients, and the average age was 61.22 ± 11.02 years. No significant differences were observed in the gender and age composition of the two groups (Table 1). The blood pressure and blood lipid indexes of the two groups are also matched with no significant differences. APE patients showed a higher level of D-D than DVT patients (*P* < 0.0001). Increasing D-D could distinguish APE patients from DVT patients (AUC = 0.702), but the specificity (64.18%) and sensitivity (67.15%) were relatively low (Figure S2).

**Table 1:** Baseline information of study subjects

	DVT	APE	*P*-value
Age	60.48 ± 8.77	61.22 ± 11.02	0.631
Gender	35/32	72/65	0.966
BMI	24.46 ± 2.32	24.27 ± 2.94	0.655
SBP	119.11 ± 9.70	120.79 ± 8.58	0.209
DBP	77.80 ± 7.99	80.50 ± 8.99	0.192
TC	4.25 ± 0.81	4.36 ± 1.03	0.456
TG	1.49 ± 0.86	1.56 ± 0.93	0.586
HDL	1.49 ± 0.33	1.53 ± 0.33	0.407
LDL	2.69 ± 0.62	2.72 ± 0.72	0.813
D-D	4.37 ± 1.34	5.51 ± 1.72	< 0.0001

BMI: body mass index, kg/m2; SBP: systolic blood pressure, mmHg; DBP: diastolic blood pressure, mmHg; TC: total cholesterol, mmol; TG: triglyceride, mmol/L; HDL: high-density lipoprotein, mmol/L; LDL: low-density lipoprotein, mmol/L; D-D: D-dimer, mg/L

### Expression and significance of mir-150-5p in APE patients

**Fig. 1:**
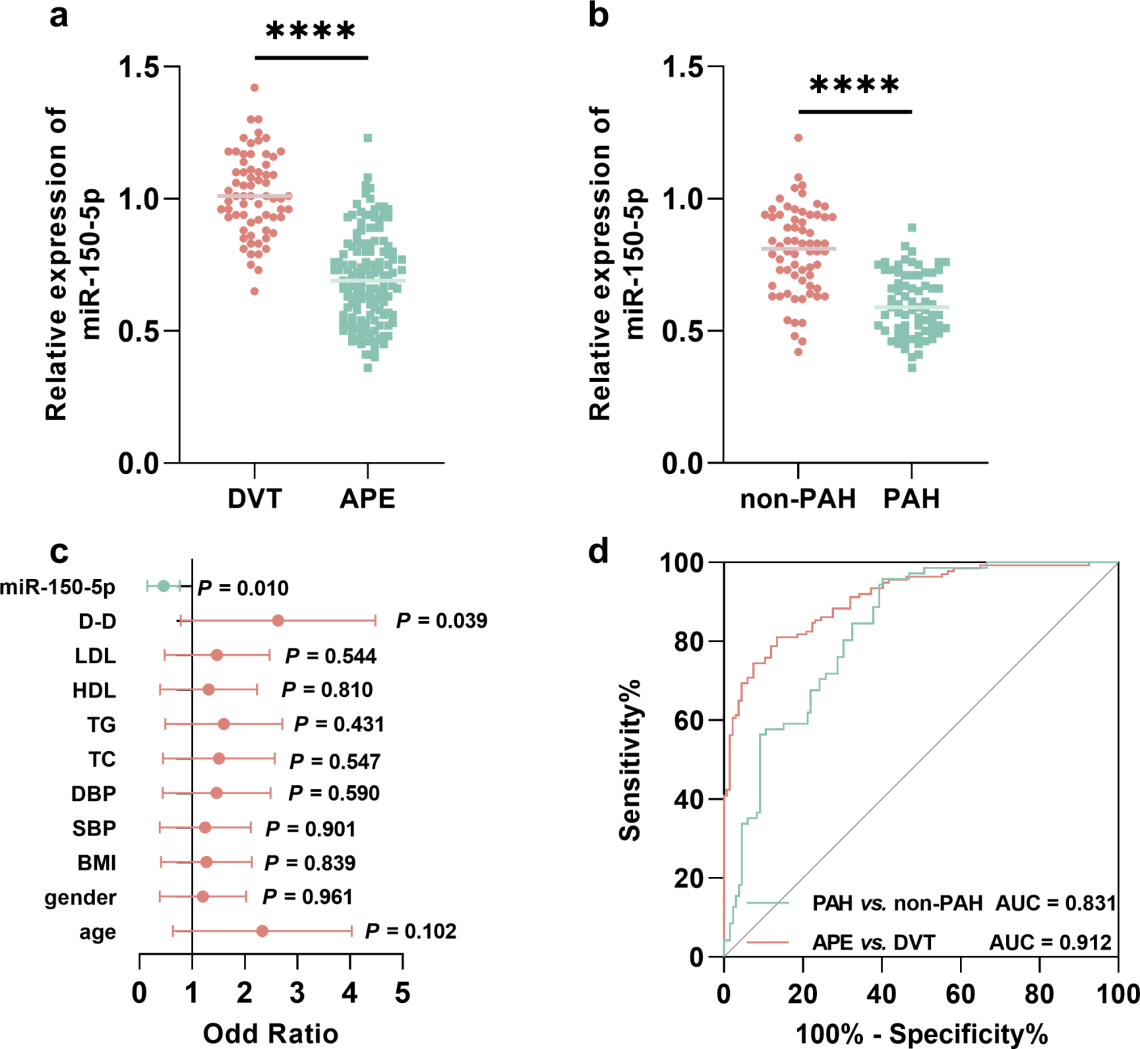
Expression and significance of miR-150-5p in APE. a-b. expression of miR-150-5p in APE (**a**) and APE patients with or without PAH (**b**). c-d. risk factor assessment for the occurrence of PAH in APE patients (**c**) and diagnostic value of miR-150-5p in APE and PAH (**d**). ^****^P < 0.0001

Compared with DVT patients, miR-150-5p was significantly downregulated in the plasma of APE patients (Fig. 1a). Based on the occurrence of pulmonary atrial hypertension (PAH), APE patients were further divided into PAH and non-PAH groups. The serum miR-150-5p level was much lower in APE patients with PAH than patients without PAH (Fig. 1b), and decreasing miR-150-5p (OR = 0.385, 95% CI = 0.186–0.798, *P* = 0.010) was identified as a risk factor for PAH as well as D-D (OR = 2.202, 95% CI = 1.040–4.663, *P* = 0.039, Fig. 1c).

miR-150-5p could distinguish APE patients from DVT patients (AUC = 0.912, 95% CI = 0.872–0.950) and could discriminate between APE patients with PAH and patients without PAH (AUC = 0.831, 95% CI = 0.762-0.900, Fig. 1d).

### Effect of mir-150-5p on the biological function of HUVECs induced by ox-LDL

**Fig. 2:**
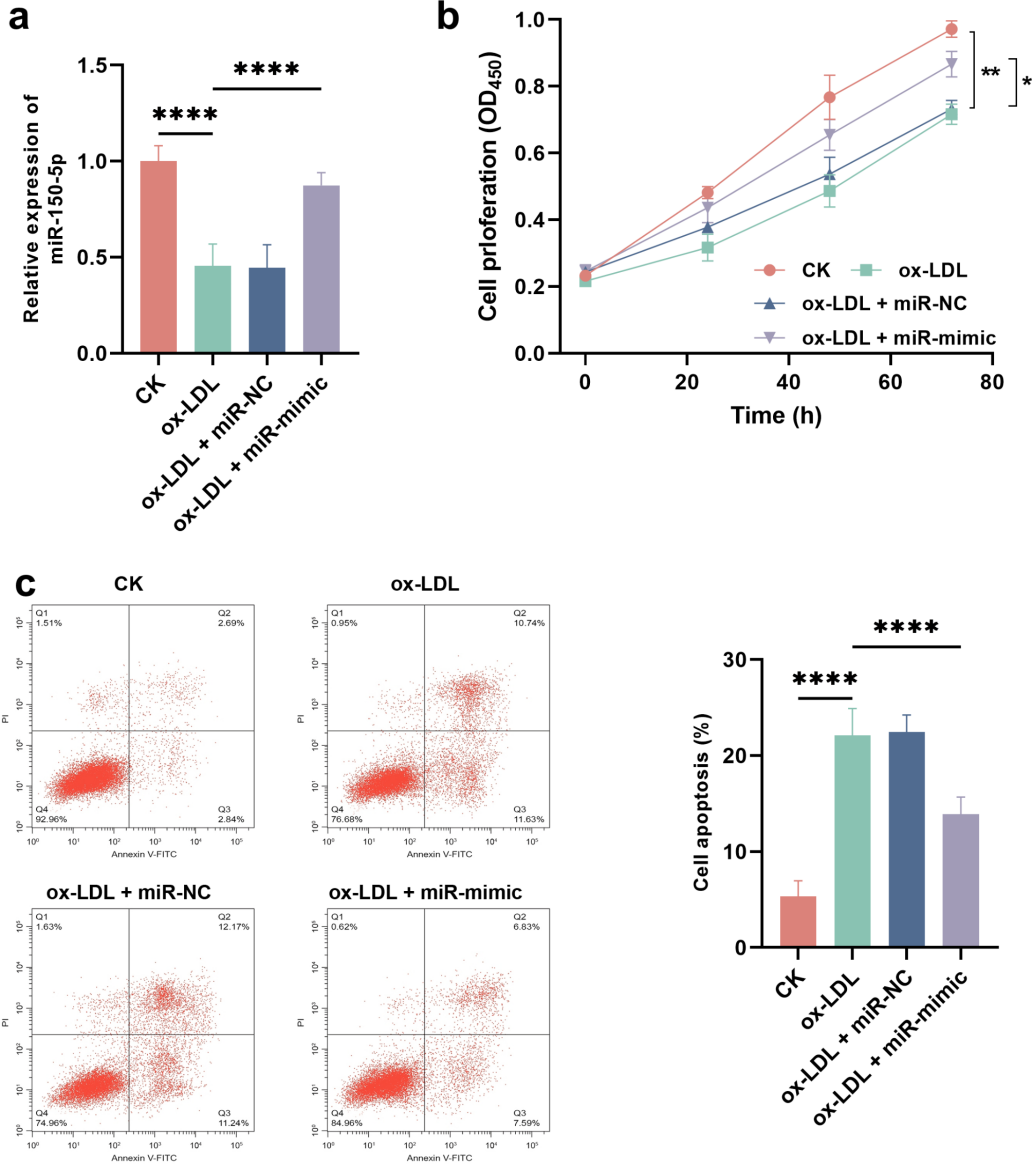
Regulatory effect of miR-150-5p on HUVECs (*n* = 3). a. expression of miR-150-5p in HUVECs treated with ox-LDL and transfected with miR-150-5p mimic. b-c. effect of miR-150-5p overexpression on cell proliferation (**b**) and apoptosis (**c**) of HUVECs. ^*^P < 0.05, ^**^P < 0.01, ^****^P < 0.0001

ox-LDL induced significant downregulation of miR-150-5p in HUVECs, which was increased by the transfection of miR-150-5p mimic (Fig. 2a). ox-LDL suppressed the proliferation (Fig. 2b) and promoted apoptosis (Fig. 2c) of HUVECs, while the overexpression of miR-150-5p could alleviate the effect of ox-LDLD on HUVECs.

### Effect of mir-150-5p on ox-LDL-induced inflammation and oxidative stress in HUVECs

**Fig. 3:**
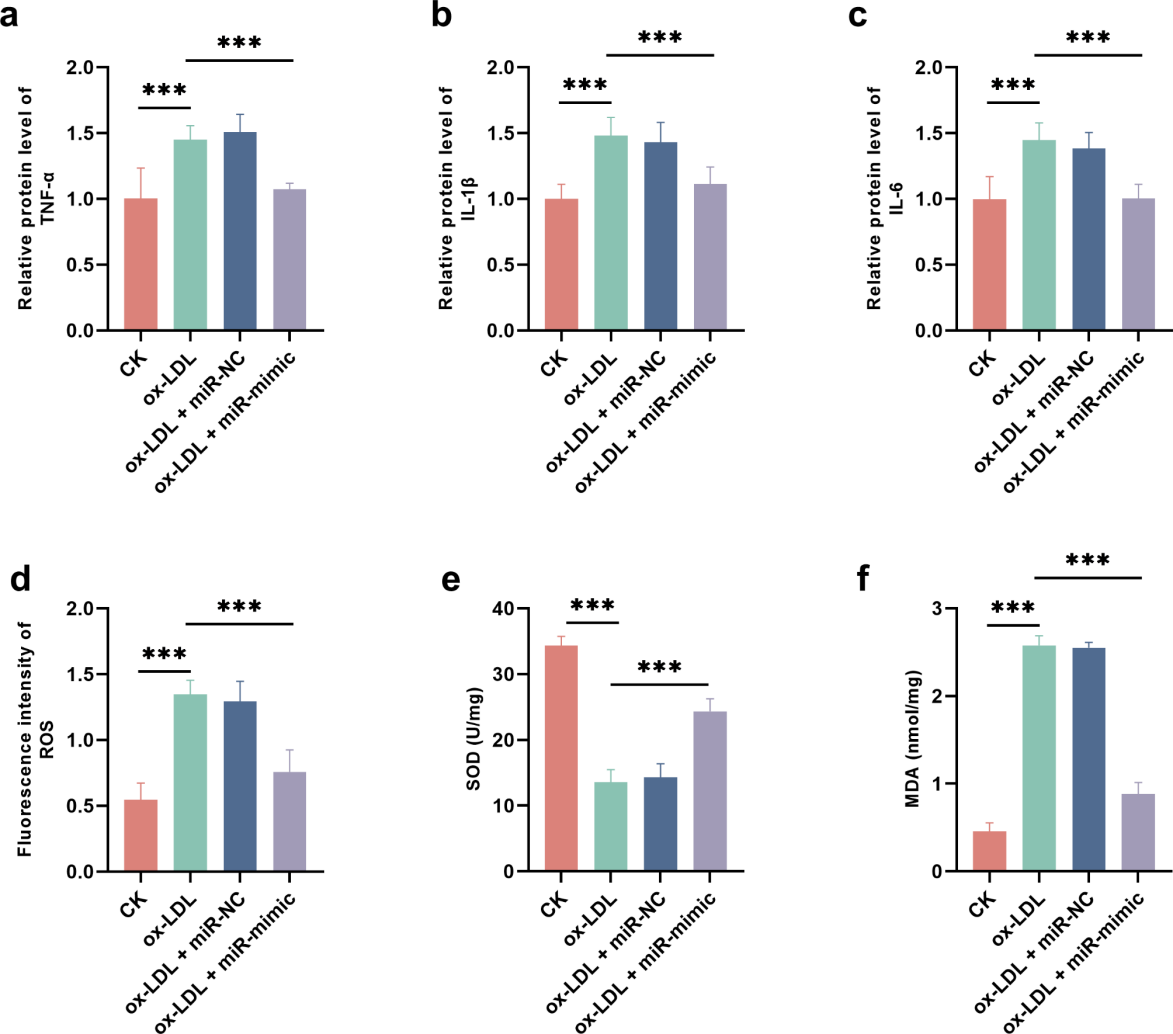
Regulatory effect of miR-150-5p on ox-LDL-induced inflammation and oxidative stress in HUVECs (*n* = 3). a-b. effect of miR-150-5p on pro-inflammatory cytokines, TNF-α (**a**), IL-1β (**b**), and IL-6 (**c**). d-e. effect of miR-150-5p on oxidative stress indicators, ROS (**d**), SOD (**e**), and MDA (**f**). ^***^P < 0.001

ox-LDL induced significant inflammation behaving as the increasing protein levels of TNF-α (Fig. 3a), IL-1β (Fig. 3b), and IL-6 (Fig. 3c). ox-LDL-induced increasing ROS (Fig. 3d), decreasing SOD (Fig. 3e), and elevating MDA (Fig. 3f) in HUVECs, indicating the significant oxidative stress. The overexpression of miR-150-5p could attenuate ox-LDL-induced inflammation (Fig. 3a-c) and oxidative stress (Fig. 3d-f) in HUVECs.

## Discussion

The similar clinical symptoms of APE with cardiopulmonary diseases increase the difficulty of APE diagnosis. It has been demonstrated that effective early detection of APE could improve the survival rate, but overdiagnosis might induce the risk of bleeding complications related with anticoagulation therapy [15]. Therefore, the accuracy, sensitivity, and specificity of APE diagnosis are of great significance. Plasma D-D was previously considered a major biomarker for the diagnosis of APE [16]. However, the specificity and sensitivity were relatively low. Therefore, D-D is mainly applied for the exclusion of APE patients or needs the combination with other clinical examinations [17]. With increasing attention to miRNAs, this study evaluated the significance of miR-150-5p in APE and revealed its significant downregulation in APE patients, which distinguishes APE patients from DVT patients with relatively higher sensitivity and specificity than D-D. Previous studies always includes healthy individuals as the control group to demonstrate the diagnostic value of miRNAs [18–20]. As APE is one of the most common complications of DVT, this study enrolled a group of DVT patients as the control group, which could help identify risk factor for APE. Future studies would also consider the enrollment of healthy individuals to evaluate the significance of miR-150-5p in screening APE. During the onset of APE, a thrombus would block the pulmonary artery, combined with the contraction of the pulmonary artery by neurohumoral factors and hypoxia, resulting in a high incidence of PAH [21, 22]. In the enrolled APE patients, over half of patients occurred PAH. APE patients occurred PAH showed a lower plasma miR-150-5p level than the patients without the complication. Moreover, reducing miR-150-5p was identified as a risk factor for PAH in APE patients and also showed a diagnostic significance to distinguish APE patients with PAH. Hence, miR-150-5p was considered a biomarker for the diagnosis and severeity of APE and the occurrence of PAH in APE patients. The demonstrated significance of miR-150-5p in APE and its complication of PAH implied that miR-150-5p is of great potential in serving as an indicator for the onset and severity of APE and could assist its clinical screnning and management, but the findings needs further clinical validations. To exclude interferencing factors as much as possible, this study exclude patients with complications, which might narrow the clinical applicability of miR-150-5p. The enrolled study subjects come from single research center increasing the risk of selection bias and limiting the generalizability of the findings Therefore, future studies should expanded the diversity of study subjects from multiple research centers to clear the significance of miR-150-5p in broader APE and DVT patients.

Endothelial cell injury is a major pathogenesis of the formation of thrombosis, which could activate the clotting process and therefore increase the risk of APE [23, 24]. Ox-LDL has been demonstrated to induce endothelial cell apoptosis and has been employed for the construction of injured cell models [25]. Ox-LDL could induce the production of ROS and induced inflammatory factors, which caused inflammatory cascade amplification response [26–28]. Consistently, ox-LDL induced significant inflammation and oxidative stress in HUVECs significantly inhibited HUVECs proliferation and promoted cell apoptosis, indicating the successful modeling of injured HUVECs. The regulation of endothelial cell injury by miRNAs has been widely reported and has been considered the potential mechanism underlying their involvement in APE development. For example, miR-28-3p regulated cell apoptosis of pulmonary endothelial cells and further promoted the progression of PE in a mouse model [29]. miR-150-5p has been identified as a thromboembolism-related miRNA and could regulate endothelial inflammation [9, 11, 14]. Herein, miR-150-5p was found to reverse the suppressed proliferation and promoted apoptosis of HUVECs by ox-LDL. Additionally, miR-150-5p also alleviated ox-LDL-induced inflammation and oxidative stress in HUVECs, indicating the involvement of miR-150-5p in endothelial cell injury during APE.

## Conclusions

In conclusion, downregulated miR-150-5p distinguished APE patients and predicted the risk of PAH in APE patients. Overexpressing miR-150-5p attenuates ox-LDL-induced endothelial cell apoptosis, inflammation, and oxidative stress, which was hypothesized the regulatory mechanism underlying the function of miR-150-5p in APE. Future studies should deeply investigate the regulatory mechanism underlying miR-150-5p to provide more reference for the therapy of APE. Moreover, except for cell modeling, animal modeling is also a reliable method to reveal the significance of miRNAs in human diseases. Therefore, further APE animal modeling should be performed to deeply understanding the function and mechanism of miR-150-5p in APE.

## Electronic supplementary material

Below is the link to the electronic supplementary material.

Supplementary Material 1

Supplementary Material 2

## Data Availability

The datasets used and/or analysed during the current study are available from the corresponding author on reasonable request.
