# Supplementary figures and images for: Mir-150-5p distinguishes acute pulmonary embolism, predicts the occurrence of pulmonary arterial hypertension, and regulates ox-LDL-induced endothelial cell injury
Source: Hereditas. 2024 Sep 10;161:33. doi: 10.1186/s41065-024-00333-z (PMC11384695; doi:10.1186/s41065-024-00333-z)

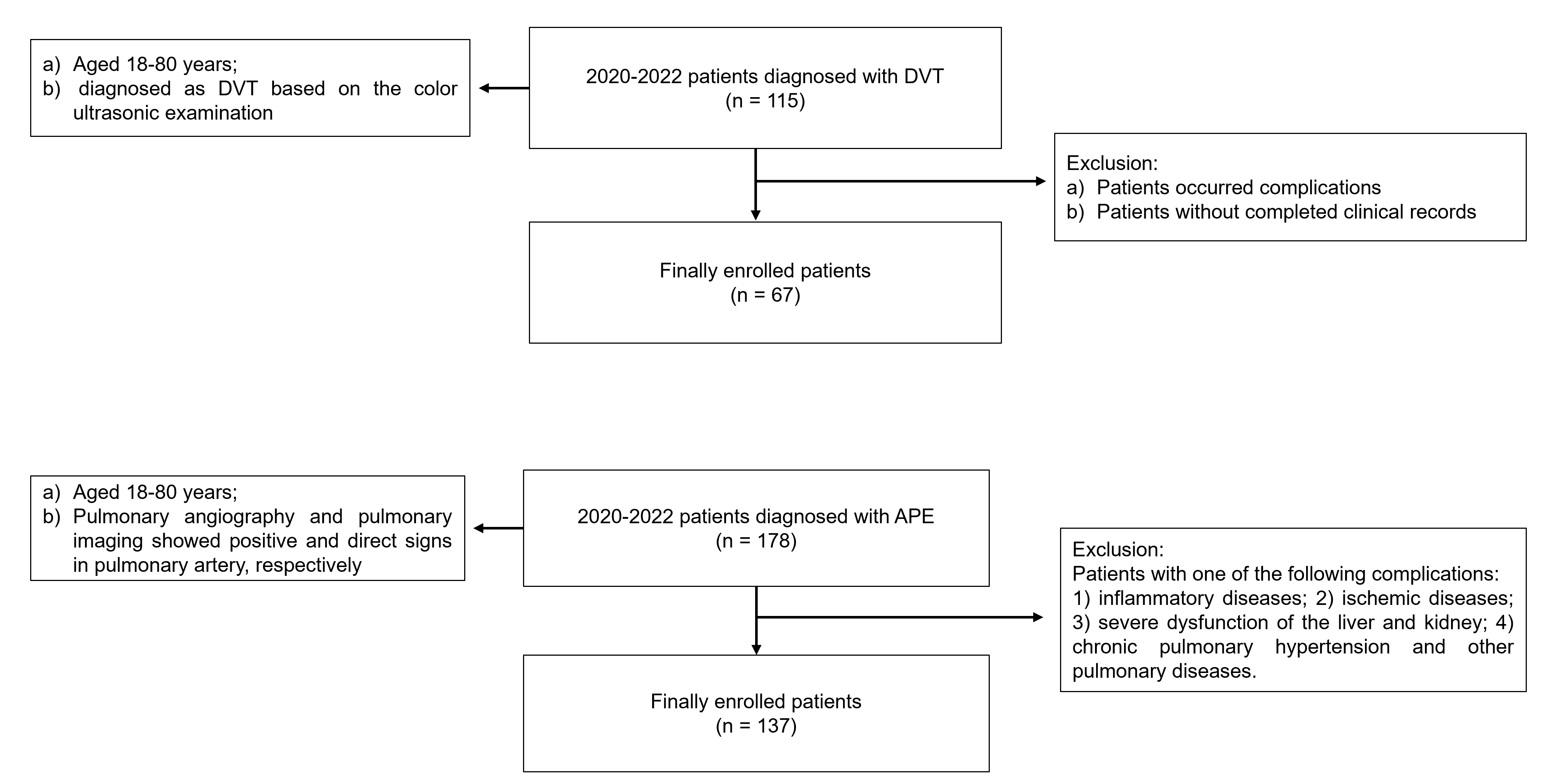

Supplement: Supplementary file 1 — Supplementary Material 1 [file 41065_2024_333_MOESM1_ESM.tif]

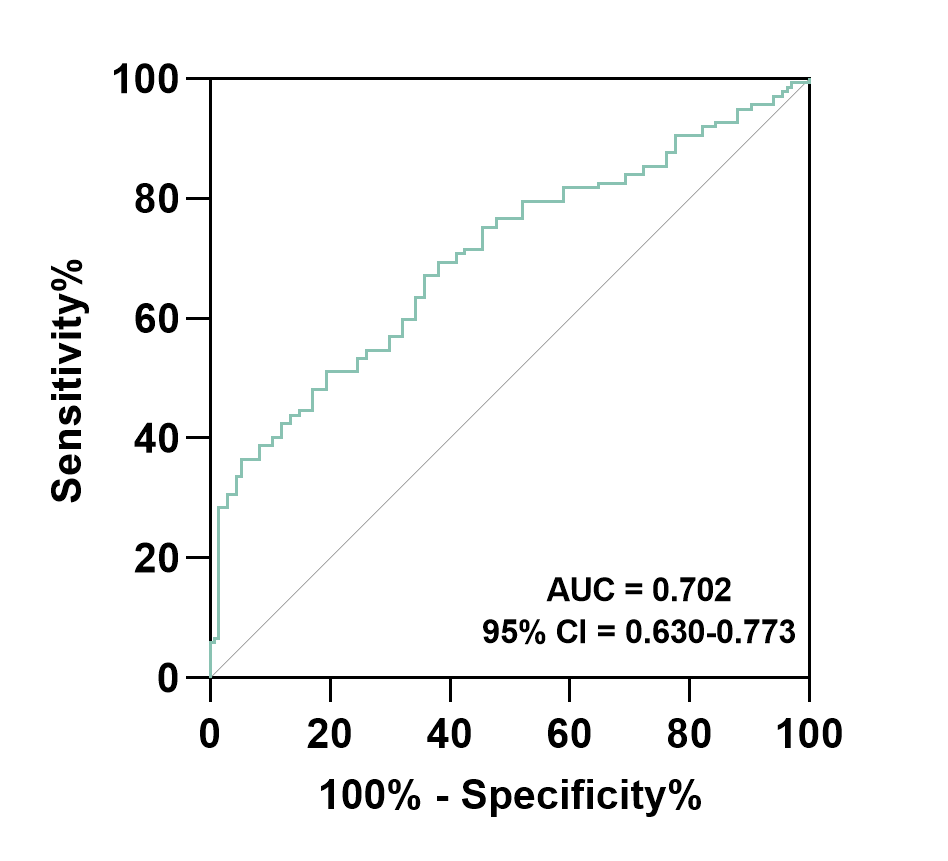

Supplement: Supplementary file 2 — Supplementary Material 2 [file 41065_2024_333_MOESM2_ESM.tif]
